# Hydrogen attenuates endothelial glycocalyx damage associated with partial cardiopulmonary bypass in rats

**DOI:** 10.1371/journal.pone.0295862

**Published:** 2023-12-19

**Authors:** Hiroki Iwata, Takasumi Katoh, Sang Kien Truong, Tsunehisa Sato, Shingo Kawashima, Soichiro Mimuro, Yoshiki Nakajima

**Affiliations:** 1 Department of Anesthesiology and Intensive Care, Hamamatsu University School of Medicine, Hamamatsu, Japan; 2 Department of Anesthesiology and Surgical Critical Care, Pham Ngoc Thach University of Medicine, Ho Chi Minh City, Vietnam; 3 Institute for Physiological Sciences, Justus-Liebig-University, Giessen, Germany; The Open University, UNITED KINGDOM

## Abstract

Cardiopulmonary bypass (CPB) causes systemic inflammation and endothelial glycocalyx damage. Hydrogen has anti-oxidant and anti-inflammatory properties; therefore, we hypothesized that hydrogen would alleviate endothelial glycocalyx damage caused by CPB. Twenty-eight male Sprague–Dawley rats were randomly divided into four groups (n = 7 per group), as follows: sham, control, 2% hydrogen, and 4% hydrogen. The rats were subjected to 90 minutes of partial CPB followed by 120 minutes of observation. In the hydrogen groups, hydrogen was administered via the ventilator and artificial lung during CPB, and via the ventilator for 60 minutes after CPB. After observation, blood collection, lung extraction, and perfusion fixation were performed, and the heart, lung, and brain endothelial glycocalyx thickness was measured by electron microscopy. The serum syndecan-1 concentration, a glycocalyx component, in the 4% hydrogen group (5.7 ± 4.4 pg/mL) was lower than in the control (19.5 ± 6.6 pg/mL) and 2% hydrogen (19.8 ± 5.0 pg/mL) groups (*P* < 0.001 for each), but it was not significantly different from the sham group (6.2 ± 4.0 pg/mL, *P* = 0.999). The endothelial glycocalyces of the heart and lung in the 4% hydrogen group were thicker than in the control group. The 4% hydrogen group had lower inflammatory cytokine concentrations (interleukin-1β and tumor necrosis factor-α) in serum and lung tissue, as well as a lower serum malondialdehyde concentration, than the control group. The 2% hydrogen group showed no significant difference in the serum syndecan-1 concentration compared with the control group. However, non-significant decreases in serum and lung tissue inflammatory cytokine concentrations, as well as in serum malondialdehyde concentration, were observed. Administration of 4% hydrogen via artificial and autologous lungs attenuated endothelial glycocalyx damage caused by partial CPB in rats, which might be mediated by the anti-inflammatory and anti-oxidant properties of hydrogen.

## Introduction

Cardiopulmonary bypass (CPB) is an essential cardiovascular procedure. However, CPB causes systemic inflammation, which is characterized by extensive vascular endothelial activation and diffuse endothelial dysfunction, and is known to impair the endothelial glycocalyx [1–5]. The glycocalyx is a layer that covers the surface of vascular endothelial cells [6, 7]. It has various functions, including vascular permeability regulation and inflammatory response suppression [6, 7]. Degradation of the glycocalyx exposes adhesion molecules in the blood vessel lumen and initiates reactions between neutrophils and the vascular endothelium [6–9]. The blood concentration of syndecan-1, a component of the glycocalyx, is related to glycocalyx degradation and correlates with the concentration of inflammatory cytokines in the blood [6, 7]. Impaired glycocalyx is associated with an exacerbated inflammatory response [6, 7, 10] and persistent microcirculatory perfusion disturbances after CPB [3]; therefore, protecting the glycocalyx is important in CPB.

Hydrogen selectively scavenges reactive oxygen species (ROS) and has anti-oxidant, anti-inflammatory, and anti-apoptotic properties [11, 12]. But it does not inhibit the action of ROS necessary for physiological activity [13]. Although it is flammable, hydrogen is safe at concentrations of <4.1% in both air and oxygen [14]. The therapeutic effect of hydrogen has been verified in various pathological conditions in animals [11, 12, 15–21]. With regard to CPB, Fujii et al. reported that administration of 1.4% hydrogen via an artificial lung restrained the inflammatory reaction during CPB in rats [22]. However, it is not clear whether hydrogen alleviates endothelial glycocalyx damage caused by CPB.

We aimed to elucidate the effects of hydrogen on endothelial glycocalyx damage in a rat model of CPB. We hypothesized that hydrogen would alleviate endothelial glycocalyx damage caused by CPB. To test this hypothesis, we evaluated the serum syndecan-1 concentration and endothelial glycocalyx thickness in the heart, lung, and brain by electron microscopy.

## Materials and methods

This study was approved by the Ethics Committee for Animal Experiments and the Laboratory Animal Facility of Hamamatsu University School of Medicine (approval no: 2020029 and 2023017). The study conformed to the Guide for the Care and Use of Laboratory Animals. Male Sprague–Dawley rats (12 to 13 weeks old, weighing 380.2 to 404.3 g; SLC Inc., Shizuoka, Japan) were used in this study. All surgeries and procedures were performed under isoflurane anesthesia, and every effort was made to minimize pain. Animals were housed at a controlled temperature (24°C) with a 12–12 h light–dark cycle, with free access to water and food.

### Animal preparation

Twenty-eight rats were randomly assigned to four groups based on a simple randomization method (n = 7 per group), as follows: sham group, control group, 2% hydrogen group, and 4% hydrogen group. The sham operation consisted of venous and arterial cannulation and heparinization without CPB. The animals were anesthetized with 5.0% isoflurane (Mylan, Tokyo, Japan). After adequate anesthesia was achieved, the rats were intubated with a 14-gauge intravenous catheter (B Braun, Melsungen, Germany) and mechanically ventilated using a small-animal ventilator (Shinano Seisakusho, Tokyo, Japan) with an air–oxygen mixture (fraction of inspired oxygen = 0.4). The respiratory rate was 60 breaths/min, tidal volume was 8 mL/kg, and positive end-expiratory pressure was 3 cmH_2_O. During surgery and CPB, anesthesia was maintained with 2% isoflurane to achieve adequate depth of anesthesia. All surgeries were performed with standard sterile techniques. Rectal temperature was monitored and regulated at 37.0°C ± 0.2°C using heat lamps and forced-air convective systems.

The tail artery was cannulated with a 22-gauge intravenous catheter (B Braun) as the arterial inflow cannula for CPB. The right femoral artery was cannulated with a 22-gauge intravenous catheter (B Braun) for arterial blood pressure monitoring and blood gas analysis. The right external jugular vein was cannulated with a modified 15-gauge intravenous catheter (Hakko Medical Device Division, Nagano, Japan) as the venous outflow for CPB. Heparin (200 IU) was administered through the venous cannula.

The CPB circuit consisted of a venous cannula, a blood reservoir (5 mL drip chamber), a custom-made roller pump (RZ1030 peristaltic pump head, Nanjing Runze Fluid Control Equipment, Nanjing, China; stepper motor, controlled by a one-chip microcomputer), an artificial lung (Senko Medical Co., Ltd., Osaka, Japan), and an arterial inflow cannula. It was primed with 10 mL Voluven (hydroxyethyl starch 130000, Otsuka Pharmaceutical Co., Ltd., Tokyo, Japan). At the top of the reservoir, an 18-gauge needle (B Braun) was inserted and connected with a custom-made negative pressure generator consisting of a peristaltic pump head, stepper motor, pressure sensor, and one-chip microcomputer.

### Experimental protocol

After surgical cannulation and heparinization, the animals were connected to the CPB circuit. After 15 minutes of stabilization, the negative pressure generator was activated to create a negative pressure in the venous reservoir, and extracorporeal circulation was slowly initiated. The negative pressure was gradually increased, and the roller pump speed was increased to a flow rate of 100 mL/kg/min (approximately 60% of cardiac output in rats [23]). Once this flow rate was reached, the respiratory rate was reduced to 20 breaths/min, and the fraction of inspired oxygen was changed to 1.0. CPB was performed for 90 minutes. Gas flow to the artificial lung consisted of 100% oxygen at 400 mL/min with 2% isoflurane. No rats received additional injections during CPB. At CPB completion, the respiratory rate was increased to 60 breaths/min, and the rats were weaned from CPB. Before disconnecting from the CPB circuit, the blood volume in the reservoir was adjusted to the same as the initial volume. Then, the right external jugular vein and tail artery catheters were removed. The rats were followed for an additional 120 minutes under general anesthesia. No rats required infusion during the observation. Rats in the hydrogen groups received selected concentrations of hydrogen from the start to 1 hour after the end of CPB. Hydrogen was supplied by HGE-1A (Shimadzu Corp., Kyoto, Japan) and administered via the ventilator and artificial lung during CPB and via the ventilator after CPB. The production rate was controlled in relation to the total fresh gas flow to obtain the desired hydrogen concentration.

Mean arterial pressure, heart rate, and body temperature were monitored continuously. They were recorded before the initiation of CPB (baseline), 45 and 90 minutes into CPB, and 60 and 120 minutes after CPB. Arterial blood gas analysis was performed at the same time points (ABL90 FLEX, Radiometer Medical ApS, Brønshøj, Denmark).

### Sample collection

At completion of observation, all rats underwent thoracotomy under anesthesia. The lower lobe of the right lung was ligated and removed. A 22-gauge catheter (B Braun) was inserted into the inferior vena cava, and 6 mL of blood was drawn. Then, the left lung was removed by clamping the hilum. After an incision was made in the right atrium, a 21-gauge needle (Nipro, Osaka, Japan) was inserted through the cardiac apex and phosphate-buffered saline was perfused (MP-2000, Tokyo Rikakai Co., Ltd., Tokyo, Japan) at 8 mL/min for 2 minutes to wash out blood from the whole body. Subsequently, fixative solution (2% glutaraldehyde, 30 mM HEPES buffer, and 2% lanthanum nitrate) was perfused for 5 minutes at a rate of 8 mL/min. After perfusion, the right lung, heart, and brain were excised. The upper lobe of the right lung, left ventricular myocardium, and cerebral cortex were cut out and immersed in fixing/staining solution for 24 hours at 4°C. After proper tissue processing, ultrathin sections were made.

Serum was collected by centrifugation at 1,000 *× g* at 4°C for 20 minutes using Centrifuge 5425 R (Eppendorf, Hamburg, Germany) and stored at −80°C until use. The inferior portion of the left lung was measured, and RIPA buffer containing protease cocktail inhibitors (Nacalai Tesque, Kyoto, Japan) was added at a 5% (w/v) ratio. It was homogenized using the Physcotron handheld homogenizer (Microtec Nition, Chiba, Japan) for 1 minute and Power Sonic model 50 (Yamato Scientific Co., Ltd., Tokyo, Japan) for 5 minutes, before centrifuging at 13,000 × *g* for 15 minutes at 4°C (Centrifuge 5425 R, Eppendorf). The supernatant was collected and stored at −80°C until analysis.

#### Glycocalyx degradation

Glycocalyx degradation was measured using enzyme-linked immunosorbent assay (ELISA) kits for serum syndecan-1 (Cloud-Clone Corp., Katy, TX, US).

#### Endothelial glycocalyx thickness

Endothelial glycocalyx in the heart, lungs, and brain was imaged by transmission electron microscopy (JEM-1400 Plus; JEOL, Tokyo, Japan). Ten perfused vessels were randomly selected for each organ and images were captured. The area of lanthanum staining and the vessel circumference were measured, and the ratio of these two measurements was defined as the average glycocalyx thickness [24].

#### Inflammation

The concentrations of interleukin-1β (IL-1β) and tumor necrosis factor-α (TNFα) in the serum and lung tissues were measured by ELISA (R&D Systems Inc., Minneapolis, MN, US).

#### Oxidative stress

Serum malondialdehyde (MDA) was measured by ELISA (Cell Biolabs Inc., San Diego, CA, US). MDA is a final product of lipid metabolism caused by oxidative stress, and the level of MDA is said to be correlated with free radical-induced damage [25].

#### Lung histology

The upper part of the left lung was embedded in paraffin, cut into 3-μm sections, and stained with anti-myeloperoxidase (MPO) antibody (Abcam, Cambridge, UK). The slides were digitized using a NanoZoomer S60 (Hamamatsu Photonics K.K., Hamamatsu, Japan). The ratio of MPO-positive cells to total cells in the sections was determined using HALO (Indica Labs, Albuquerque, New Mexico, US) and used as a measure of neutrophil activation.

#### Lung wet-to-dry weight ratio

Lung wet-to-dry weight ratio was used as a measure of pulmonary edema. The right lower lobe was weighed immediately after excision, and the dry weight was recorded after drying the tissue in an oven at 75°C for 72 hours.

### Statistical analysis

The power analysis was performed using G* power software (G* Power 3.1.9.7, University of Düsseldorf, Germany) to determine the required number of specimens. Based on our report on the glycocalyx-protecting action of hydrogen [17], the effect size of d was 1.52. Assuming a sample size ratio of 1 and a statistical power of 1 − β = 0.8 to identify significant differences (α = 0.05), 7 animals were required per group.

The primary outcome was the serum syndecan-1 concentration, and the secondary outcomes were endothelial glycocalyx thickness in the heart, lung, and brain; molecular biology results; and immunohistochemistry results. Variables were tested for normality with the Shapiro–Wilk test. Normally distributed data are expressed as the mean ± standard deviation and non-normally distributed data as the median (interquartile range [25th-75th percentile]). For comparisons between the four groups, parametric data were analyzed using one-way analysis of variance followed by the Tukey–Kramer multiple-comparisons test. The Kruskal–Wallis test followed by the Steel–Dwass multiple-comparisons test was used when data were non-normally distributed. *P* < 0.05 (two-tailed) was considered statistically significant. Statistical analyses were performed using JMP for Windows (version 14.2.0, SAS Institute Inc., NC, US).

## Results

### Physiologic measurements

The body weights of the sham, control, 2% hydrogen, and 4% hydrogen groups were 394.3 ± 7.9 g, 392.5 ± 6.4 g, 391.2 ± 7.1 g, and 396.3 ± 3.1 g, respectively. No significant differences were observed among the four groups. The physiologic values are detailed in Table 1. The data were similar for all groups at the baseline. During CPB, hematocrit was lower in the three groups that underwent CPB than in the sham group. Lactate was lower in the sham group than in the other three groups. At the end of observation, mean arterial pressure in the sham group was higher than the other three groups. Heart rate was lower in the 4% hydrogen group than in the 2% hydrogen and control groups, and was not significantly different from the sham group. Lactate was lower in the 4% hydrogen group than in the control group and was not significantly different from the sham group.

**Table 1:** Physiologic values.

		CPB		Post-CPB	
	Baseline	45min	90min	60min	120min
**MAP (mmHg)**					
sham	84.4 ± 5.7	79.2 ± 8.4	77.7 ± 7.9	80.1 ± 6.2	81.0 ± 5.8
control	86.7 ± 6.3	80.8 ± 9.9	80.1 ± 9.8	65.1 ± 13.0	48.2 ± 11.0**
2% hydrogen	90.7 ± 5.2	83.1 ± 6.3	77.5 ± 12.9	66.1 ± 12.9	56.0 ± 8.3**
4% hydrogen	87.0 ± 7.0	98.5± 8.3**##††	95.4 ± 8.1*#†	66.5 ± 12.3	58.0 ± 11.1**
**HR (beat/min)**					
sham	332.1 ± 19.4	327.1 ± 10.6	316.1 ± 22.9	302.8 ± 20.0	302.2 ± 20.6
control	348.5 ± 18.1	337.8 ± 4.4	351.2 ± 11.6	367.4 ± 30.4**	352.7 ± 25.7**
2% hydrogen	342.1 ± 45.9	325.1 ± 15.1	320.2 ± 44.3	349.0 ± 49.7	348.8 ± 27.2**
4% hydrogen	337.1 ± 22.5	334.0 ± 6.2	352.5 ± 26.2	316.4 ±24.5#	305.5 ± 20.9##†
**Hct (%)**					
sham	39.2 ± 1.9	38.1 ± 1.4	37.5 ± 0.8	36.6 ± 0.8	35.6 ± 1.1
control	39.6 ± 1.0	25.1 ± 1.5**	26.6 ± 1.4**	27.9 ± 1.1**	26.7 ± 1.2**
2% hydrogen	39.5 ± 1.2	23.6 ± 0.8**	24.8 ± 0.9**	25.7 ± 1.1**#	24.7 ± 1.4**
4% hydrogen	39.5 ± 1.4	25.5 ± 1.3**	27.2 ± 1.8**†	28.2 ± 1.9**†	27.8 ± 2.1**††
**pH**					
sham	7.45 ± 0.02	7.44 ± 0.03	7.42 ± 0.02	7.41 ± 0.01	7.40 ± 0.03
control	7.45 ± 0.02	7.53 ± 0.01**	7.49 ± 0.01**	7.38 ± 0.03	7.38 ± 0.02
2% hydrogen	7.45 ± 0.02	7.55 ± 0.04**	7.52 ± 0.04**	7.39 ± 0.01	7.40 ± 0.00
4% hydrogen	7.43 ± 0.02	7.49 ± 0.02†	7.47 ± 0.02*	7.39 ± 0.02	7.38 ± 0.02
PaO_2_ (mmHg)					
sham	186.2 ±19.9	185.7 ± 19.1	180.2 ± 21.0	183.0 ± 14.1	190.2 ± 17.2
control	176.8 ± 12.9	210.8 ± 34.5	166.4 ± 33.9	181.5 ± 15.9	191.7 ± 15.6
2% hydrogen	181.7 ± 13.7	181.4 ± 32.7	130.2 ± 28.3*	185.7 ± 15.5	204.2 ± 15.3
4% hydrogen	186.2 ± 18.8	211.4 ± 36.8	170.2 ± 45.7	197.1 ± 16.5	203.2 ± 16.0
PaCO_2_ (mmHg)					
sham	36.6 ± 2.2	36.4 ± 2.5	37.3 ± 2.7	37.6 ± 1.9	38.5 ± 2.5
control	37.2 ± 2.8	28.6 ± 2.1**	29.5 ± 3.9**	37.6 ± 2.7	36.0 ± 2.9
2% hydrogen	37.5 ± 1.3	27.4 ± 4.1**	26.9 ± 4.8**	36.2 ± 1.8	35.0 ± 2.7
4% hydrogen	39.8 ± 2.3	32.9 ± 2.1#††	33.0 ± 2.1†	38.4 ± 2.7	39.1 ± 3.4
HCO_3_^-^ (mmol/L)					
sham	25.7 ± 1.2	24.9 ± 0.8	24.3 ± 1.0	23.9 ± 1.1	24.0 ± 1.4
control	25.9 ± 1.2	24.0 ± 1.5	21.8 ± 2.0*	22.2 ± 1.7	21.5 ± 2.1*
2% hydrogen	26.2 ± 1.2	23.8 ± 1.3	21.6 ± 1.3**	22.3 ± 0.9	22.1 ± 1.5
4% hydrogen	27.0 ±1.3	25.1 ± 1.0	24.1 ± 1.0#†	23.4 ± 1.2	22.7 ± 1.2
**Lac (mmol/L)**					
sham	1.9 ± 0.3	1.5 ± 0.3	1.6 ± 0.4	1.7 ± 0.6	2.0 ± 0.4
control	2.0 ± 0.2	2.0 ± 0.6	3.5 ± 1.2**	4.1 ± 1.0**	4.7 ± 1.2**
2% hydrogen	2.0 ± 0.3	2.3 ± 0.4*	3.6 ± 0.5**	3.6 ± 0.9**	3.8 ± 0.8**
4% hydrogen	2.5 ± 0.4*	2.4 ± 0.4**	3.0 ± 0.5**	3.1 ± 0.4*	2.8 ± 0.5##
**Rectal temperature (°C)**					
sham	37.0 ± 0.1	37.0 ± 0.1	37.0 ± 0.1	36.9 ± 0.1	36.9 ± 0.1
control	37.0 ± 0.2	37.0 ± 0.0	37.0 ± 0.1	37.0 ± 0.1	37.0 ± 0.0
2% hydrogen	36.9 ± 0.1	37.1 ± 0.0	36.9 ± 0.0	37.0 ± 0.1	36.9 ± 0.1
4% hydrogen	37.0 ± 0.0	37.0 ± 0.1	36.9 ± 0.1	37.0 ± 0.0	36.9 ± 0.1

Data are presented as the mean ± standard deviation of 7 rats per group. Comparisons were performed by one-way analysis of variance with Tukey–Kramer’s multiple-comparisons post hoc test (*P* < 0.05).

*P < 0.05

**P < 0.01; versus sham group.

#*P* < 0.05

##*P* < 0.01; versus control group.

†*P* < 0.05

††*P* < 0.01; versus 2% hydrogen group. CPB, cardiopulmonary bypass; MAP, mean arterial blood pressure; HR, heart rate; Hct, hematocrit; Lac, lactate.

### Endothelial glycocalyx degradation after CPB

**Fig 1:**
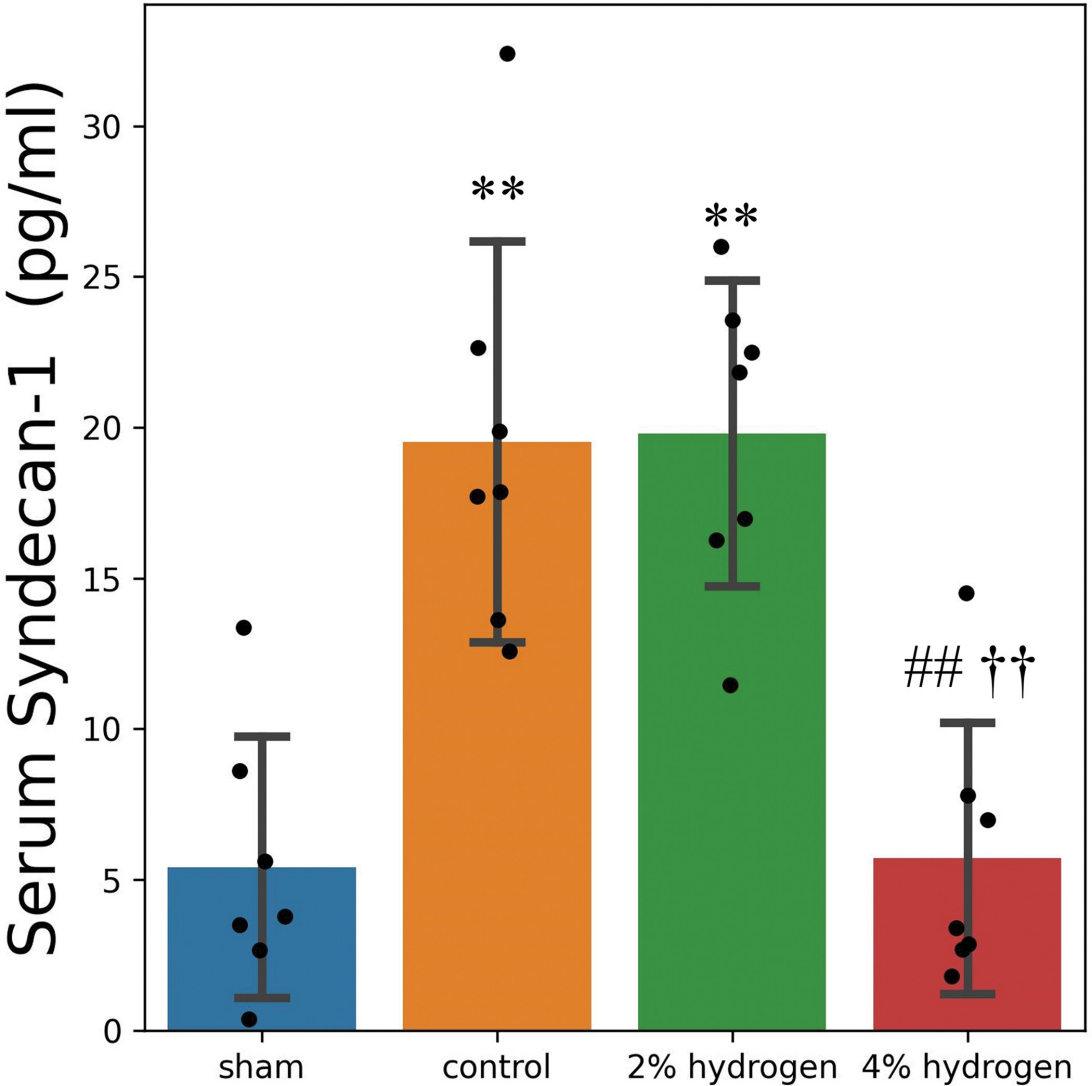
Serum syndecan-1 concentration. Data are presented as the mean ± standard deviation of 7 rats per group. Comparisons were performed by one-way analysis of variance with Tukey–Kramer’s multiple-comparisons post hoc test (*P* < 0.05). **P < 0.01 versus sham group. ##*P* < 0.01 versus control group. ††*P* < 0.01 versus 2% hydrogen group.

Serum syndecan-1 in the 4% hydrogen group was lower than in the control and 2% hydrogen groups (*P* < 0.001 for each), and was not significantly different from the sham group (*P* = 0.999). There was no significant difference between the 2% hydrogen and control groups (*P* = 0.999), and both groups demonstrated higher syndecan-1 concentrations than the sham group (*P* < 0.001 for each) (Fig 1).

#### Endothelial glycocalyx thickness in the heart, lung, and brain after CPB

**Fig 2:**
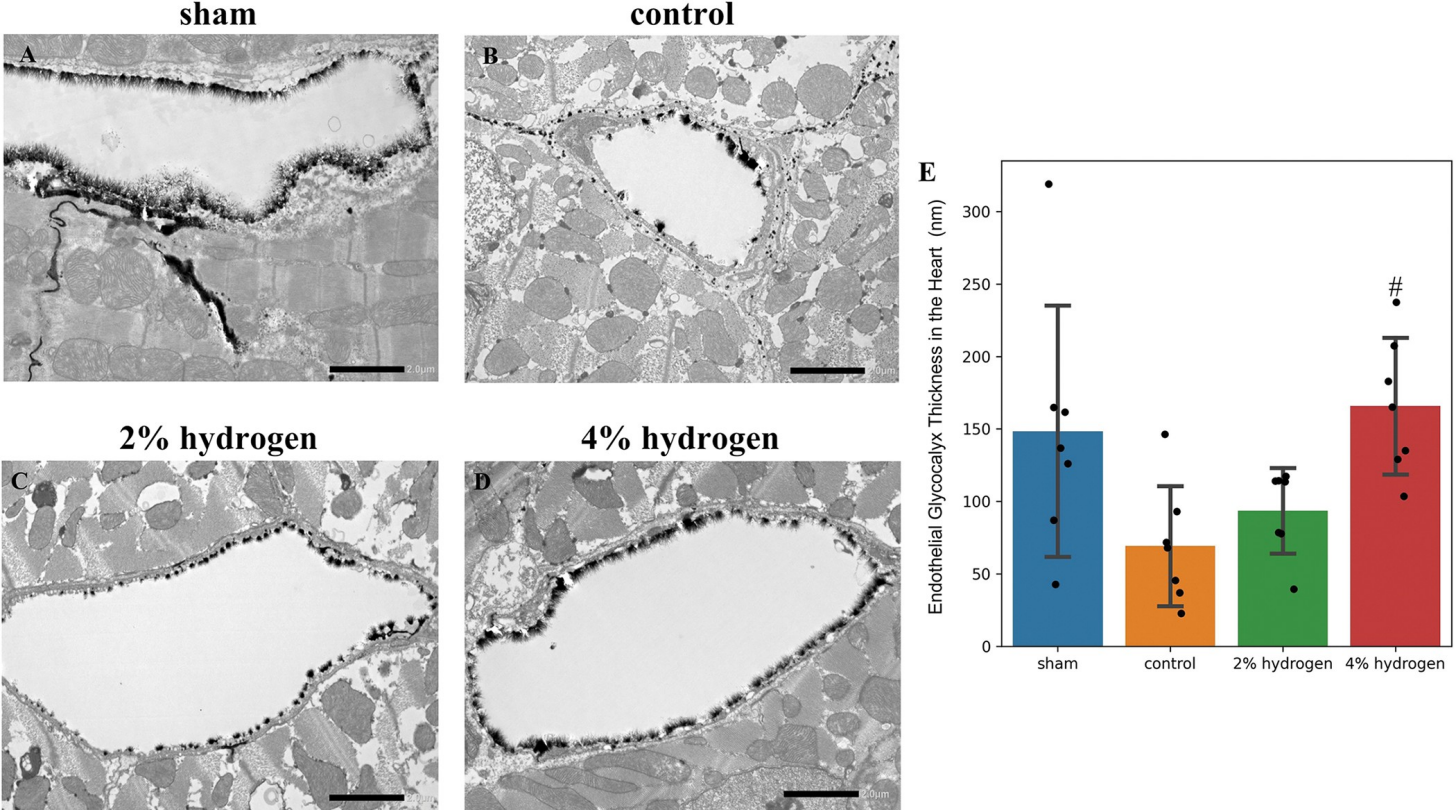
Impairment of the endothelial glycocalyx in the heart by CPB and the effects of hydrogen. (A–D) Representative transmission electron microscopy images of the endothelial glycocalyx in the heart. The endothelial glycocalyx was visualized by staining with lanthanum nitrate and is shown as the black layer that lines the luminal surface of vascular endothelial cells. Scale bar = 2.0 μm. (E) Thickness of the endothelial glycocalyx in the heart. Values are expressed as the mean ± standard deviation of 10 perfused vessels per group. Comparisons were performed by one-way analysis of variance with Tukey–Kramer’s multiple-comparisons post hoc test (*P* < 0.05). #*P* < 0.05 versus control group. CPB, cardiopulmonary bypass.

The heart endothelial glycocalyx was thicker in the 4% hydrogen group than in the control group (*P* = 0.016), and no significant difference was observed between the 2% hydrogen group and the control group (*P* = 0.843), the 2% hydrogen group and the sham group (*P* = 0.276), and the control group and the sham group (*P* = 0.060) (Fig 2).

**Fig 3:**
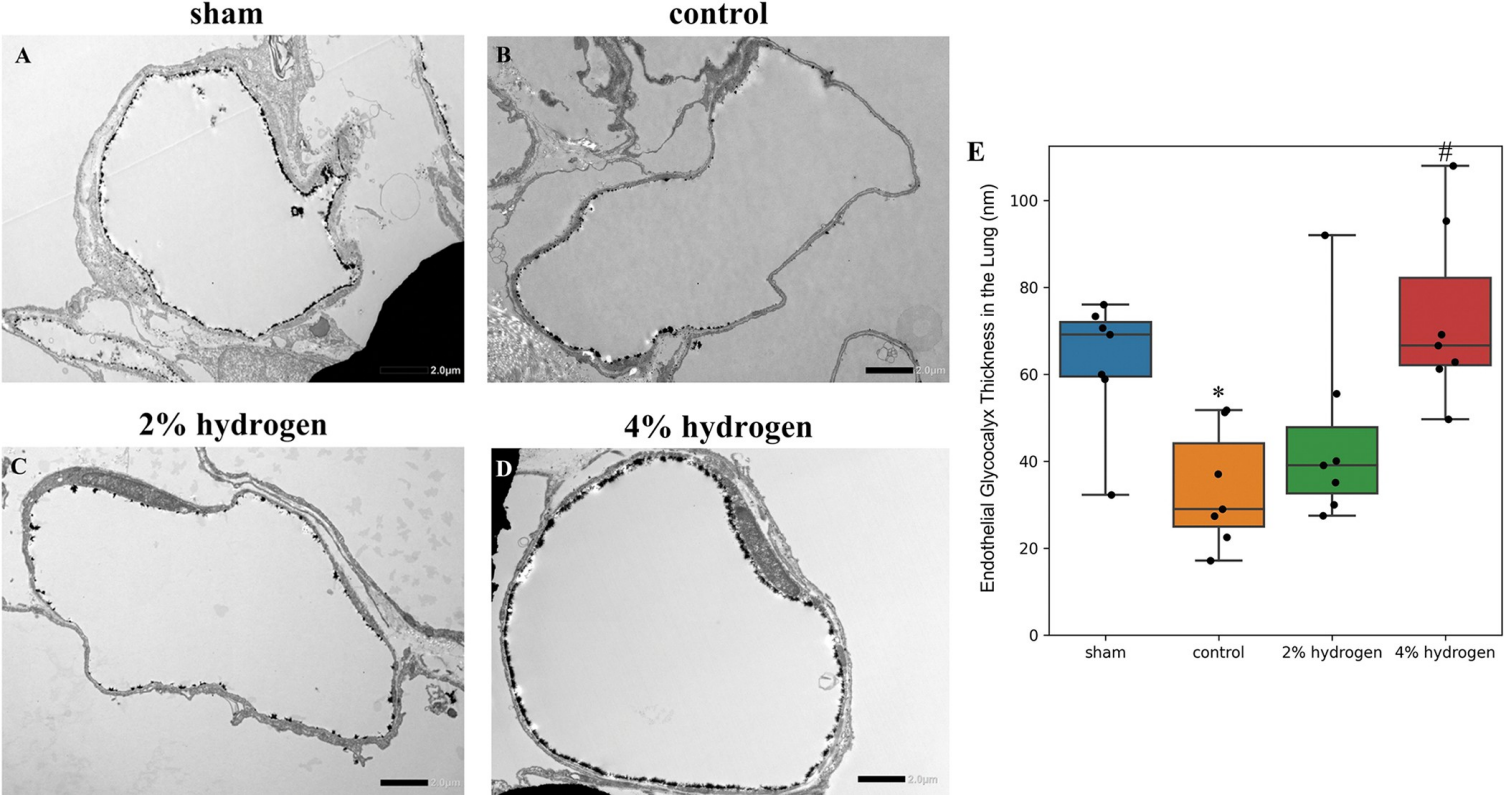
Impairment of the endothelial glycocalyx in the lung by CPB and the effects of hydrogen. (A–D) Representative transmission electron microscopy images of the endothelial glycocalyx in the lung. The endothelial glycocalyx was visualized by staining with lanthanum nitrate and is shown as the black layer that lines the luminal surface of vascular endothelial cells. Scale bar = 2.0 μm. (E) Thickness of the endothelial glycocalyx in the lung. The box plots represent the median and interquartile range of 10 perfused vessels per group. Comparisons were performed using the Kruskal–Wallis test with the Steel–Dwass multiple-comparisons post hoc test (*P* < 0.05). *P < 0.05 versus sham group. #*P* < 0.05 versus control group. CPB, cardiopulmonary bypass.

The lung endothelial glycocalyx was thicker in the 4% hydrogen group than in the control group (*P* = 0.025), and thinner in the control group than in the sham group (*P* = 0.036). No significant difference was observed between the 2% hydrogen group and the control group (*P* = 0.577) (Fig 3).

**Fig 4:**
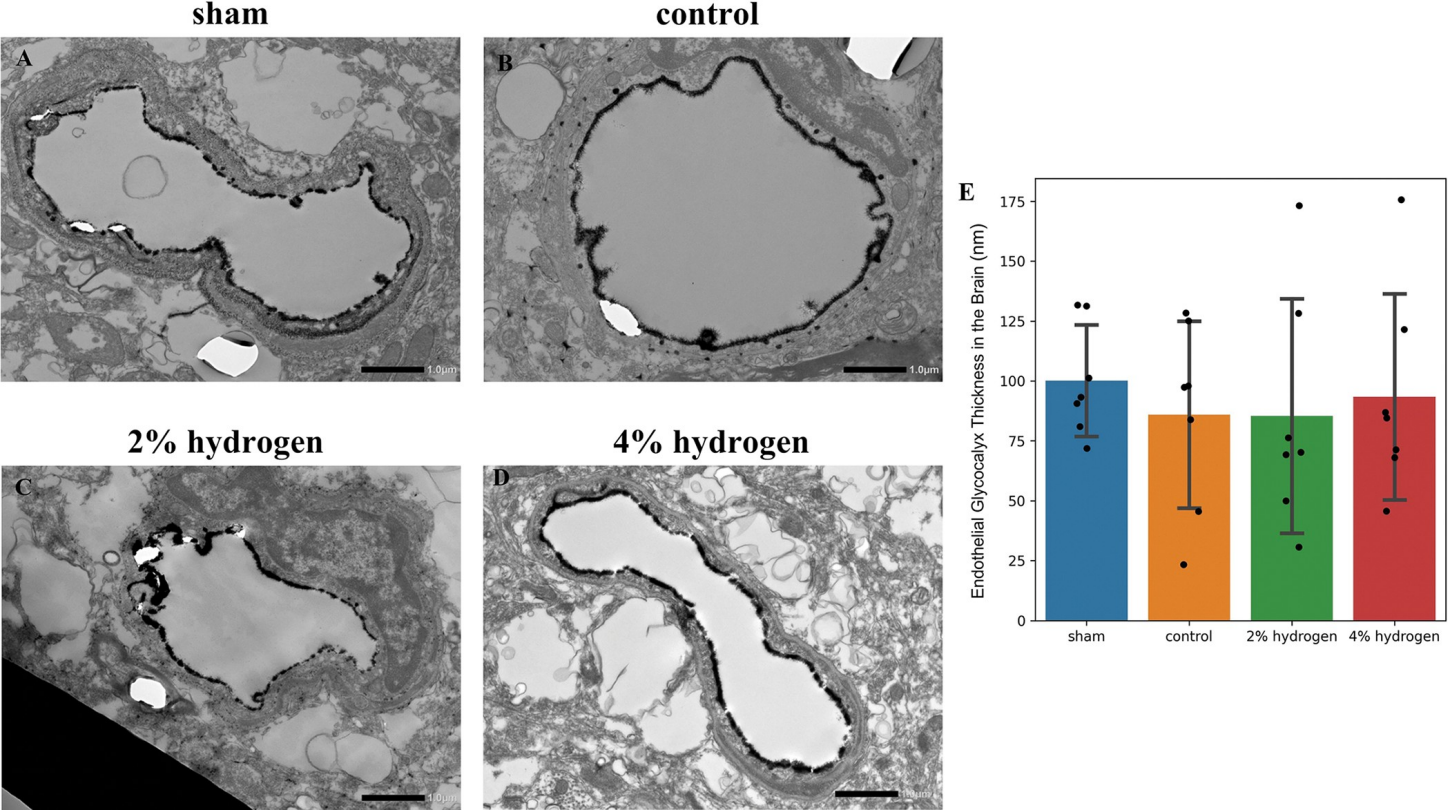
Impairment of the endothelial glycocalyx in the brain by CPB and the effects of hydrogen. (A–D) Representative transmission electron microscopy images of the endothelial glycocalyx in the brain. The endothelial glycocalyx was visualized by staining with lanthanum nitrate and is shown as the black layer that lines the luminal surface of vascular endothelial cells. Scale bar = 1.0 μm. (E) Thickness of the endothelial glycocalyx in the brain. Values are expressed as the mean ± standard deviation of 10 perfused vessels per group. Comparisons were performed by one-way analysis of variance with Tukey–Kramer’s multiple-comparisons post hoc test (*P* < 0.05). There were no significant differences among the four groups. CPB, cardiopulmonary bypass.

For the brain endothelial glycocalyx, there were no significant differences among the four groups (*P* = 0.884) (Fig 4).

### Serum inflammatory cytokines and oxidative stress

**Fig 5:**
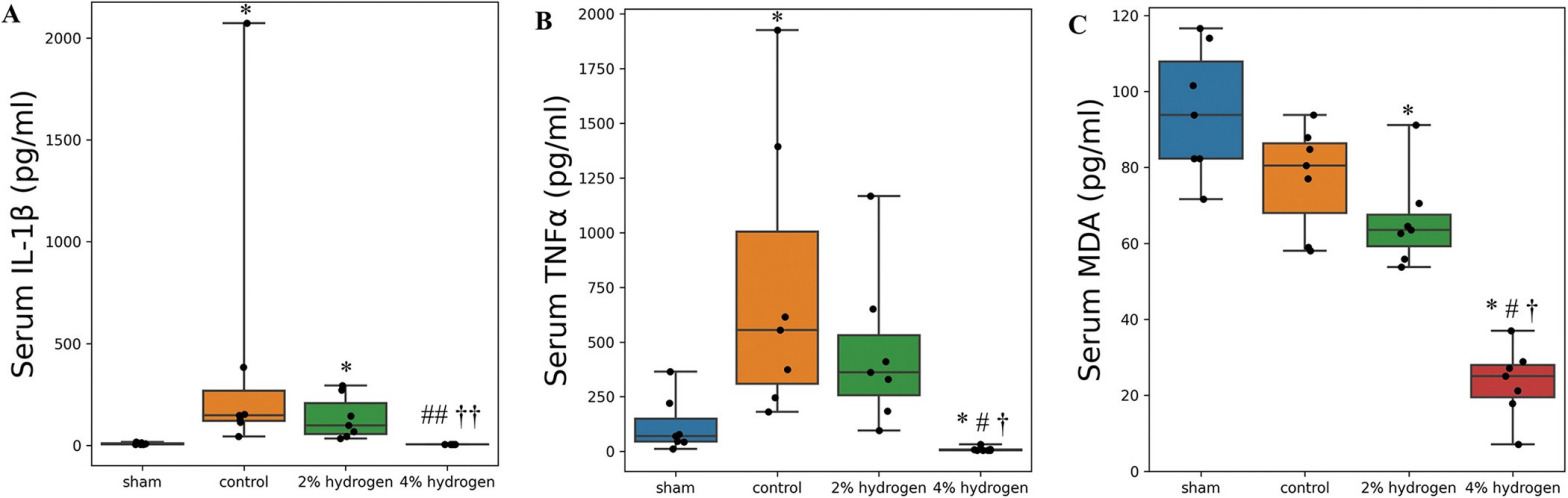
Serum concentrations of IL-1β, TNFα, and MDA. A: IL-1β. B: TNFα. C: MDA. Values are expressed as the median and interquartile range of 7 rats per group. Comparisons were performed using the Kruskal–Wallis test with the Steel–Dwass multiple-comparisons post hoc test (*P* < 0.05). *P < 0.05, **P < 0.01 versus sham group. #*P* < 0.05, ##*P* < 0.01 versus control group. †*P* < 0.05, ††*P* < 0.01 versus 2% hydrogen group. IL-1β, interleukin-1β; TNFα, tumor necrosis factor-α; MDA, malondialdehyde.

Serum IL-1β was lower than the measurable range in seven samples from the 4% hydrogen group and in three samples from the sham group. Assuming the minimum detectable value of 5.0 pg/mL by ELISA (R&D Systems Inc.), serum IL-1β was lower in the 4% hydrogen group than in the control and 2% hydrogen groups (*P* = 0.005 for each), and no significant difference to the sham group was observed (*P* = 0.133). There was no significant difference between the 2% hydrogen and control groups (*P* = 0.697), with both groups demonstrating higher IL-1β concentrations than the sham group (*P* = 0.011 for each) (Fig 5).

Serum TNFα was lower than the measurable range in four samples from the 4% hydrogen group. Assuming the minimum detectable value of 5.0 pg/mL by ELISA (R&D Systems Inc.), TNFα was lower in the 4% hydrogen group than in the control, 2% hydrogen, and sham groups (*P* = 0.010, *P* = 0.010, and *P* = 0.015, respectively). Serum TNFα was higher in the control group than in the sham group (*P* = 0.036), and was not significantly different from the 2% hydrogen group (*P* = 0.869) (Fig 5).

Serum MDA was lower in the 4% hydrogen group than in the control, 2% hydrogen, and sham groups (*P* = 0.011 for each). Serum MDA was lower in the 2% hydrogen group than in the sham group (*P* = 0.036), and was not significantly different from the control group (*P* = 0.577). No significant difference was observed between the control and sham groups (*P* = 0.378) (Fig 5).

### Lung tissue inflammation

In one animal in the control group, lung tissue contamination with fixative led to inaccurate ELISA and pathological evaluations, so lung tissue evaluation for this animal was considered as missing data.

#### Lung tissue inflammatory cytokine expression

**Fig 6:**
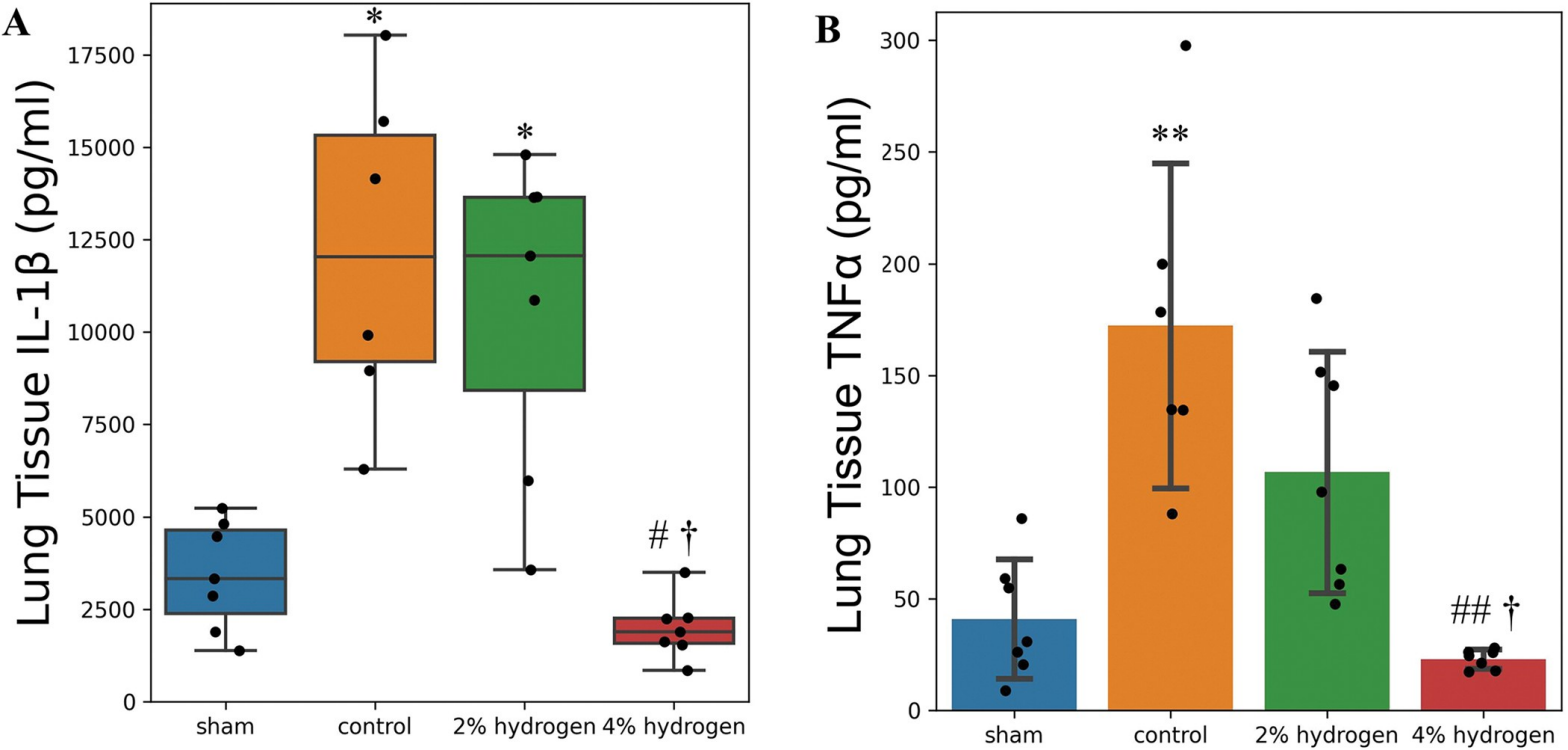
Concentration of IL-1β and TNFα in lung tissue. There were 7 animals per group, except the control group (6 animals). (A) IL-1β: Values are expressed as the median and interquartile range. Comparisons were performed using the Kruskal–Wallis test with the Steel–Dwass multiple-comparisons post hoc test (*P* < 0.05). (B) TNFα: Values are expressed as the mean ± standard deviation. Comparisons were performed using one-way analysis of variance with Tukey–Kramer’s multiple-comparisons post hoc test (*P* < 0.05). *P < 0.05, **P < 0.01 versus sham group. #*P* < 0.05, ##*P* < 0.01 versus control group. †*P* < 0.05, ††*P* < 0.01 versus 2% hydrogen group. IL-1β, interleukin-1β; TNFα, tumor necrosis factor-α.

IL-1β in the lung tissue was lower in the 4% hydrogen group than in the control and 2% hydrogen groups (*P* = 0.017 and *P* = 0.011, respectively), and no significant difference was observed compared with the sham group (*P* = 0.417). There was no significant difference between the 2% hydrogen and control groups (*P* = 0.918), and both groups demonstrated higher IL-1β concentrations than the sham group (*P* = 0.036 and *P* = 0.017, respectively) (Fig 6).

TNFα in the lung tissue was lower in the 4% hydrogen group than in the control and 2% hydrogen groups (*P* < 0.001 and *P* = 0.011, respectively), but no significant difference was observed when compared with the sham group (*P* = 0.883). The 2% hydrogen group showed no significant difference from the control group (*P* = 0.075), and TNFα was higher in the control group than in the sham group (*P* < 0.001) (Fig 6).

#### Neutrophils in lung tissue

**Fig 7:**
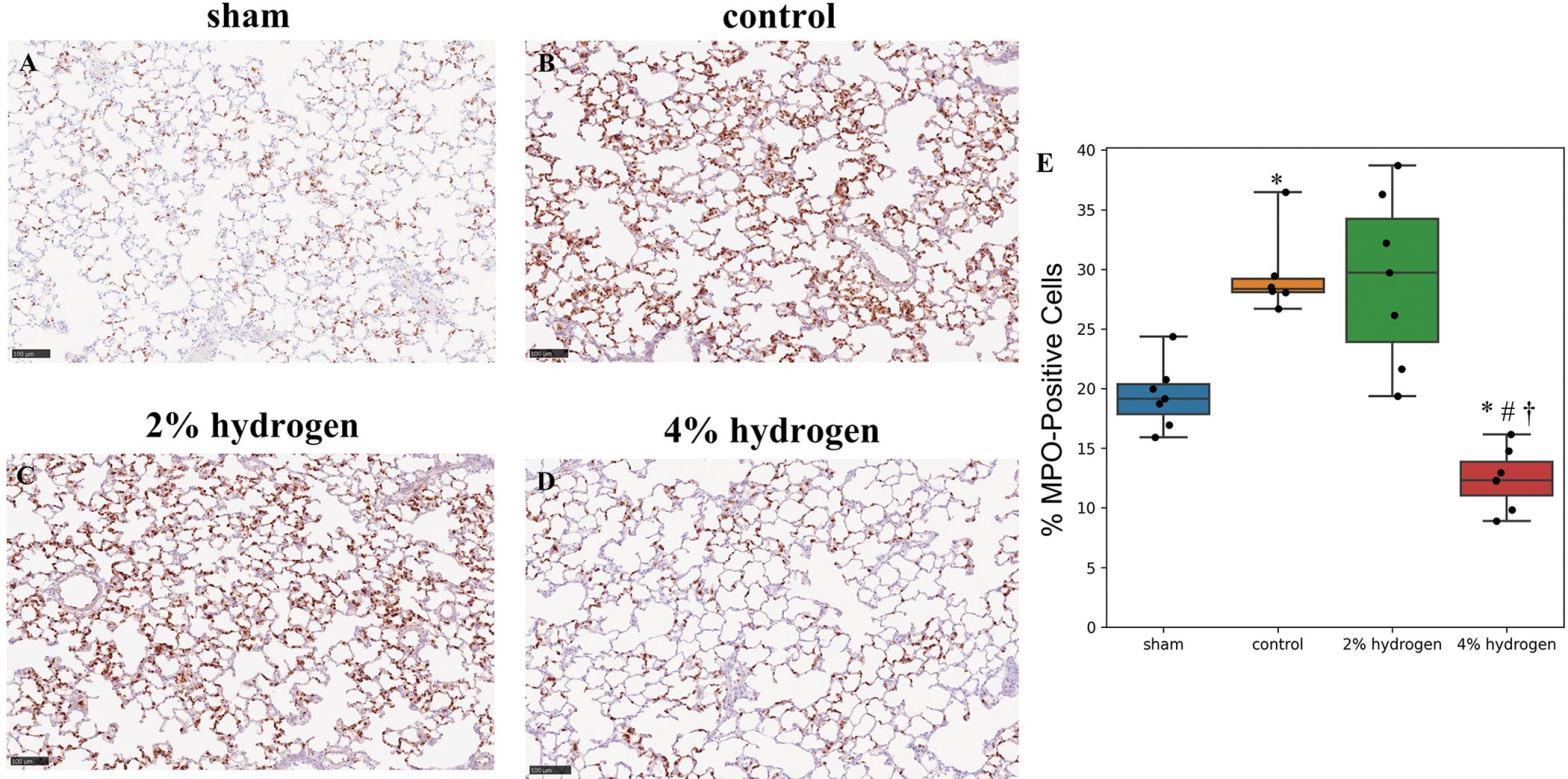
Immunohistochemical staining for MPO in lung tissue. (A-D) Photomicrographs were taken at ×200. Scale bar = 100 μm. Dark brown cells are MPO-positive cells. (E) The box plots represent the median and interquartile range of 7 animals per group, except the control group (6 animals). Comparisons were performed using the Kruskal–Wallis test followed by the Steel–Dwass multiple-comparisons post hoc test (*P* < 0.05). *P < 0.05 versus sham group. #*P* < 0.05 versus control group. †*P* < 0.05 versus 2% hydrogen group. MPO, myeloperoxidase.

The ratio of MPO-positive cells in lung tissue was lower in the 4% hydrogen group than in the control, 2% hydrogen, and sham groups (*P* = 0.017, *P* = 0.011, and *P* = 0.017, respectively). The ratio was higher in the control group than in the sham group (*P* = 0.017), and no significant difference was observed between the 2% hydrogen group and the control group (*P* = 1.000) or between the 2% hydrogen group and the sham group (*P* = 0.051) (Fig 7).

**Fig 8:**
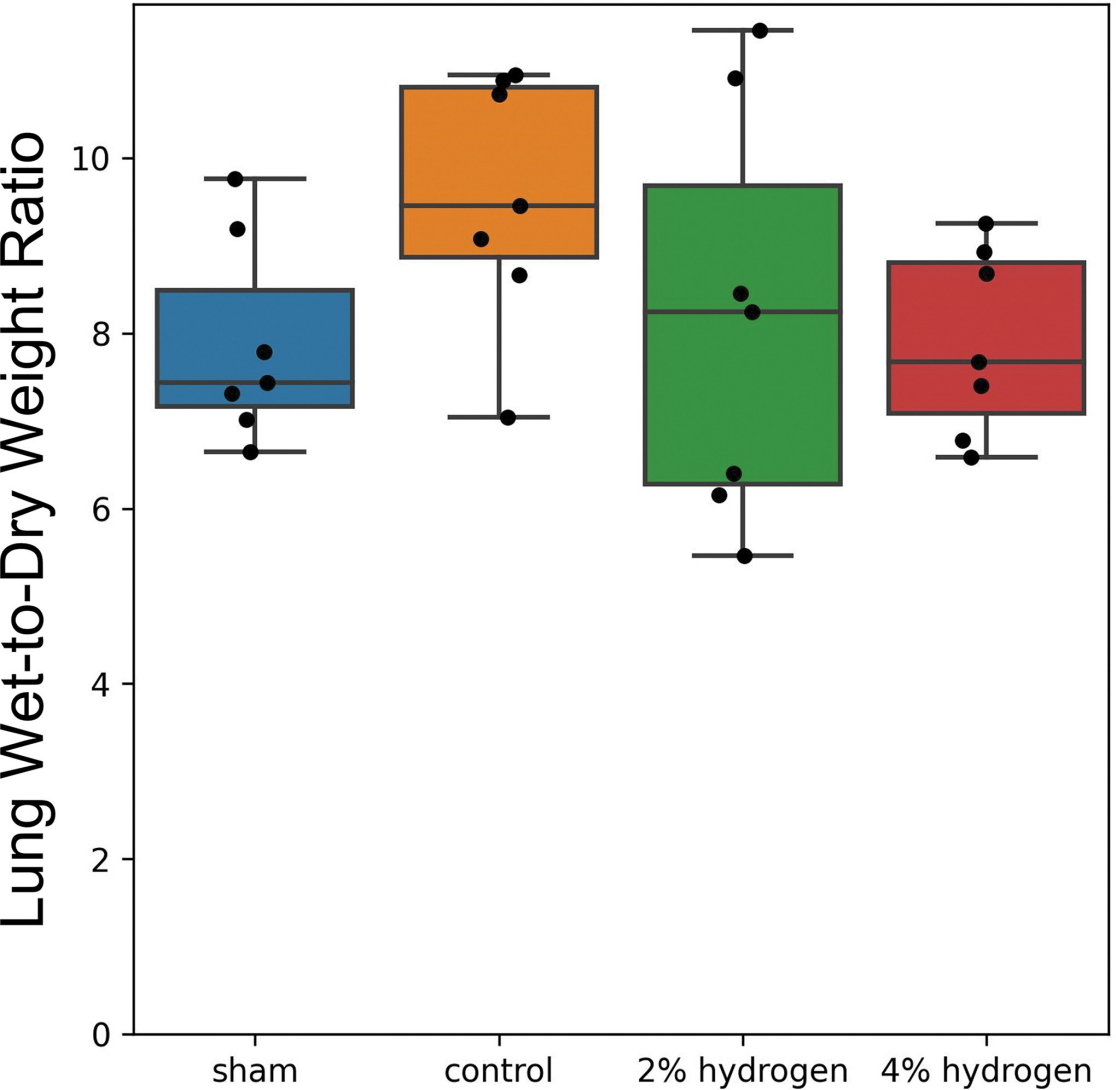
Lung wet-to-dry weight ratio. Values are expressed as the median and interquartile range of 7 animals per group. Comparisons were performed using the Kruskal–Wallis test with the Steel–Dwass multiple-comparisons post hoc test (*P* < 0.05). No significant differences were observed among the four groups.

**Lung tissue wet-to-dry ratio.** No significant differences were observed among the four groups in the lung wet-to-dry weight ratio (*P* = 0.222) (Fig 8).

## Discussion

We examined the effects of hydrogen on the endothelial glycocalyx in a rat model of partial CPB. The 4% hydrogen group had a significantly lower serum syndecan-1 concentration than the control group, indicating that 4% hydrogen attenuated endothelial glycocalyx damage. The 4% hydrogen group also had significantly lower serum IL-1β, TNFα, and MDA concentrations than the control group, indicating that 4% hydrogen had anti-inflammatory and anti-oxidant effects. In addition, the 4% hydrogen group showed significantly lower inflammatory cytokine and neutrophil levels in the lung tissue than the control group, suggesting that 4% hydrogen alleviated lung tissue inflammation.

The glycocalyx is degraded by metalloproteinases, heparinases, and hyaluronidases, which are activated by IL-1β, TNFα, and ROS [6, 26]. In this study, the 4% hydrogen group showed significantly lower syndecan-1, IL-1β, TNFα, and ROS concentrations in the serum than the control group. Thus, the protective effect of hydrogen on the glycocalyx was most likely due to anti-inflammatory and anti-oxidant effects. In addition, the lung endothelial glycocalyx in the 4% hydrogen group was thicker than in the control group, and the 4% hydrogen group showed significantly lower IL-1β, TNFα, and neutrophil expression in lung tissue than the control group. Therefore, it is suggested that preserving the lung endothelial glycocalyx alleviated the inflammatory reaction in the lung tissue in the 4% hydrogen group. In contrast, no significant differences were observed among the four groups in the wet-to-dry weight ratio of the lung. Wet lung weight includes interstitial exudate, retained intravascular blood, hemorrhage into tissues, and extravascular protein leakage; therefore, weight measurements may contain significant error [27, 28]. In addition, the 4% hydrogen group had significantly lower serum TNFα and MDA concentrations, and the ratio of MPO-positive cells in lung tissue was lower than in the sham group. Huang et al. reported that 5 hours of mechanical ventilation at normal tidal volumes induced ventilator-induced lung injury in mice [27]. This experiment was conducted with mechanical ventilation for approximately 5 hours; therefore, ventilator-induced lung injury would have occurred in the sham group. It is suggested that 4% hydrogen suppresses inflammation associated with CPB, but also long-term mechanical ventilation.

In this study, the 2% hydrogen group did not show a significant difference in serum syndecan-1 compared with the control group. This result indicated that 2% hydrogen did not attenuate damage to the endothelial glycocalyx. However, the 2% hydrogen group showed a non-significant decrease in serum IL-1β, TNFα, and MDA, as well as in IL-1β and TNFα, in lung tissue, compared with the control group. The therapeutic effect of hydrogen is dependent on the blood concentration of hydrogen [11]. When hydrogen is administered via the lungs, the blood concentration at 2% is reported to have the best therapeutic effect, and the therapeutic effect is attenuated at concentrations higher or lower than this [11]. However, in this study, 4% hydrogen was more effective than 2%, possibly due to partial CPB. During CPB, the respiratory rate was reduced to one third of normal, so hydrogen uptake through the lungs was lower than under normal respiratory conditions. Hydrogen was also administered via an artificial lung. In studies in which 2% hydrogen was administered via an artificial lung in an ischemia–reperfusion model using an extracorporeal circulatory circuit, the therapeutic effects were observed when the circuit was filled with acellular solution [29], but not when filled with blood [30]. These reports suggest that it might be difficult to take hydrogen into the blood via an artificial lung. Therefore, it is considered that the blood concentration of hydrogen during CPB in this experiment might have been lower than in the case of transtracheal hydrogen administration. In addition, humans and rodents have intestinal bacteria that produce hydrogen [31], suggesting that a certain blood concentration must be exceeded for hydrogen to have a therapeutic effect. This report suggests that the blood concentration of hydrogen in the 2% hydrogen group may have been too low to achieve an effective therapeutic effect. Both the artificial lung and the lung exchange gases by diffusion due to the gas concentration gradient, and the 4% hydrogen group seemed to take up more hydrogen than the 2% hydrogen group. Therefore, the blood concentration of hydrogen in the 4% hydrogen group was probably closer to the blood concentration at which the therapeutic effect is best achieved than in the 2% hydrogen group.

Fujii et al. reported that administration of 1.4% hydrogen via an artificial lung suppressed the inflammatory reaction during CPB [22]. This report is contrary to the results of the 2% hydrogen group in the present study. This may be related to the CPB duration, which was proportional to neutrophil adhesion molecule expression and lung injury severity [1]. The CPB duration in the present study was 1.5-times longer, suggesting that our experiment caused a higher degree of inflammation than in the previous study. If the degree of injury is too severe, it becomes difficult to obtain the therapeutic effects of hydrogen [32]. Therefore, in this experiment, inflammation may have been too severe to observe the therapeutic effects of 2% hydrogen.

In addition to the therapeutic effects of hydrogen, this experiment revealed the organ specificity of endothelial glycocalyx damage associated with CPB. The endothelial glycocalyces of the heart and lung were significantly thinner in the control group than in the sham group, whereas the brain endothelial glycocalyx was not significantly different. Our findings suggest that the brain endothelial glycocalyx is less susceptible to damage from CPB. The brain endothelial glycocalyx is one of the components of the blood–brain barrier [33]. Okamura et al. evaluated CPB-associated blood–brain barrier impairment in a piglet model and found that there was no significant difference between the CPB with normothermia group and the sham group [34], similar to our results. In addition, Ando et al. reported that in a mouse model of sepsis, endothelial glycocalyx in the brain was less disrupted than endothelial glycocalyx in the heart and lungs [35], and the present results show a similar trend. It has also been reported that bovine cerebral endothelial cells are more negatively charged than human umbilical endothelial cells [36]. This property may be the reason why the brain endothelial glycocalyx is less susceptible to damage.

### Limitations

There are several limitations in this study. First, partial CPB was performed due to artificial lung blood flow restriction. Therefore, it is unknown whether the same effect of hydrogen would be obtained with complete CPB. Second, endothelial glycocalyx evaluation by transmission electron microscopy required perfusion fixation, which precluded inflammatory response assessment in the heart and brain. Finally, the actual blood hydrogen concentration was not measured due to the relatively large blood volume required for the size of the animal.

## Conclusions

During partial CPB in rats, administration of 4% hydrogen via artificial and autologous lungs attenuated endothelial glycocalyx damage caused by CPB. Hydrogen could be a new method to protect endothelial glycocalyx by inhibiting the inflammatory response and oxidative stress derived from CPB.
